# Task-specific enhancement of hippocampus-dependent learning in mice deficient in monoacylglycerol lipase, the major hydrolyzing enzyme of the endocannabinoid 2-arachidonoylglycerol

**DOI:** 10.3389/fnbeh.2015.00134

**Published:** 2015-06-02

**Authors:** Yasushi Kishimoto, Barbara Cagniard, Maya Yamazaki, Junko Nakayama, Kenji Sakimura, Yutaka Kirino, Masanobu Kano

**Affiliations:** ^1^Laboratory of Neurobiophysics, Kagawa School of Pharmaceutical Sciences, Tokushima Bunri UniversitySanuki, Kagawa, Japan; ^2^Department of Neurophysiology, Graduate School of Medicine, The University of TokyoBunkyo-ku, Tokyo, Japan; ^3^Department of Cellular Neurobiology, Brain Research Institute, Niigata UniversityNiigata, Japan

**Keywords:** endocannabinoid, 2-Arachidonoylglycerol, monoacylglycerol lipase, hippocampus, learning and memory, extinction, morris water maze

## Abstract

Growing evidence indicates that the endocannabinoid system is important for the acquisition and/or extinction of learning and memory. However, it is unclear which endocannabinoid(s) play(s) a crucial role in these cognitive functions, especially memory extinction. To elucidate the physiological role of 2-arachidonoylglycerol (2-AG), a major endocannabinoid, in behavioral and cognitive functions, we conducted a comprehensive behavioral test battery in knockout (KO) mice deficient in monoacylglycerol lipase (MGL), the major hydrolyzing enzyme of 2-AG. We found age-dependent increases in spontaneous physical activity (SPA) in MGL KO mice. Next, we tested the MGL KO mice using 5 hippocampus-dependent learning paradigms (i.e., Morris water maze (MWM), contextual fear conditioning, novel object recognition test, trace eyeblink conditioning, and water-finding test). In the MWM, MGL KO mice showed normal acquisition of reference memory, but exhibited significantly faster extinction of the learned behavior. Moreover, they showed faster memory acquisition on the reversal-learning task of the MWM. In contrast, in the contextual fear conditioning, MGL KO mice tended to show slower memory extinction. In the novel object recognition and water-finding tests, MGL KO mice exhibited enhanced memory acquisition. Trace eyeblink conditioning was not altered in MGL KO mice throughout the acquisition and extinction phases. These results indicate that 2-AG signaling is important for hippocampus-dependent learning and memory, but its contribution is highly task-dependent.

## Introduction

The G protein-coupled receptors for Δ9-tetrahydrocannabinol—the major psychoactive chemical compound in marijuana (Cannabis sativa)—are known as cannabinoid receptors type 1 (CB_1_) and 2 (CB_2_; Gaoni and Mechoulam, 1964; Devane et al., 1988; Howlett et al., 2002). Endogenous ligands for CB_1_ receptors (endocannabinoids) function as retrograde messengers at central synapses (Kano et al., 2009). Endocannabinoids are released by the strong depolarization of postsynaptic neurons and subsequent elevation of Ca^2+^ concentration (Kreitzer and Regehr, 2001; Ohno-Shosaku et al., 2001; Wilson and Nicoll, 2001), strong activation of postsynaptic G_q/11_-coupled receptors at a basal Ca^2+^ level (Maejima et al., 2001), or by the combination of Ca^2+^ elevation and G_q/11_-coupled receptor activation (Varma et al., 2001; Ohno-Shosaku et al., 2002; Hashimotodani et al., 2005; Maejima et al., 2005). In addition, endocannabinoid signaling mediates or regulates long-term synaptic plasticity in the hippocampus (Carlson et al., 2002; Mereu et al., 2003; Chevaleyre and Castillo, 2004) and cerebellum (Safo and Regehr, 2005; van Beugen et al., 2006; Carey et al., 2011). Growing evidence indicates that the endocannabinoid system is important for various brain functions including learning and memory (Kano et al., 2009; Abush and Akirav, 2010; Morena and Campolongo, 2014). Notably, previous reports have demonstrated that CB_1_ is critical for the extinction of certain forms of memory (Marsicano et al., 2002; Varvel et al., 2005; Steinmetz and Freeman, 2011; de Bitencourt et al., 2013; Kuhnert et al., 2013).

Endocannabinoids are lipids and comprise the endogenous cannabis-like ligands that derive from arachidonic acid (Kano et al., 2009). The most thoroughly investigated endocannabinoids are N-arachidonoylethanolamine (anandamide; AEA) and 2-arachidonoylglycerol (2-AG). Endocannabinoid signaling is terminated by enzymatic hydrolysis. Anandamide is degraded by fatty acid amide hydrolase (FAAH), whereas 2-AG is hydrolyzed mainly by monoacylglycerol lipase (MGL; Cravatt et al., 1996; Dinh et al., 2002). Recent studies have clarified that not AEA but 2-AG, is the endocannabinoid that mediates retrograde signaling at central synapses (Hashimotodani et al., 2008, 2013; Gao et al., 2010; Tanimura et al., 2010). Indeed, blockade of MGL significantly induces prolonged endocannabinoid-mediated retrograde suppression of neurotransmission and altered synaptic plasticity in several brain regions (Hashimotodani et al., 2007; Pan et al., 2009; Tanimura et al., 2012; Griebel et al., 2015; Zhang et al., 2015). Although 2-AG is important for modulating synaptic transmission, relatively little is known regarding whether and how 2-AG signaling contributes to learning and memory. A previous study suggests that MGL knockout (KO) mice exhibit enhanced acquisition of hippocampus-dependent learning, including reference memory in the Morris water maze (MWM; Pan et al., 2011). However, a recent study demonstrated that pharmacological inhibition of MGL impairs hippocampal-dependent memories (Griebel et al., 2015).

The purpose of the present study was to determine the contribution of 2-AG to cognitive functions, particularly extinction learning. We examined five hippocampus-dependent learning paradigms in MGL KO mice and their heterozygous (Het) and wild-type (WT) littermates. Our results show that the effects of MGL deletion are variable and task-dependent, but indicate that 2-AG signaling is important for extinction learning in hippocampus-dependent spatial tasks.

## Materials and Methods

### Animals

A total of 166 mice were used in the present study (WT, *n* = 54; Het, *n* = 52; KO, *n* = 60). MGL KO mice were obtained on a C57BL background as described previously (Uchigashima et al., 2011). MGL KO mice were produced by homologous recombination using the embryonic stem (ES) cell line RENKA, which was derived from the C57BL/6N strain (Mishina and Sakimura, 2007). WT and Het littermates were used for comparative purposes. Genotype was confirmed by polymerase chain reaction amplification of genomic DNA extracted from the tail of each mouse, using specific primers (Uchigashima et al., 2011). The mice were housed in a room with controlled humidity, temperature, and a 12/12-h light/dark (LD) cycle with light from 09:00 to 21:00. All behavioral experiments were performed during the light phase of the LD cycle. All animal procedures were approved by Niigata University, the University of Tokyo, and Tokushima Bunri University Animal Ethics Committees, and were carried out in accordance with the guidelines laid down by the National Institutes of Health (NIH), USA. All experiments were performed by an operator who was blind to the genotype of the mice.

### Behavioral Assays

#### Timeline of the Behavioral Test Battery

**Figure 1:**
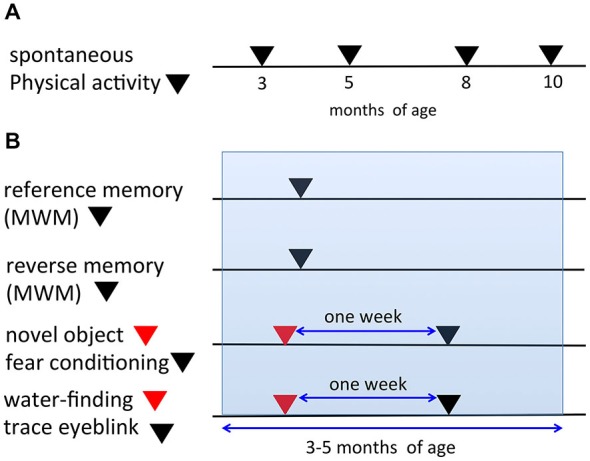
**Timeline and schematic representation for the behavioral tests. (A)** In the spontaneous physical activity (SPA) assessment, the same mice were evaluated repeatedly at 3, 5, 8, and 10 months of age. **(B)** Representation of the procedures for the six learning paradigms (reference memory of the MWM, reverse memory of the MWM, novel object recognition test, fear conditioning, water-finding test, trace eyeblink conditioning). The same mice were used for both the novel object recognition task and fear conditioning experiments with a 1-week interval in between the tests. Additionally, for the water-finding test and trace eyeblink conditioning, the same mice were used with a 1-week interval in between the tests. The ages of the mice during the six learning paradigms fell within 3–5 months of age (blue range).

In the present study, we used the same mice for certain tasks. Figure 1 depicts the timeline for all behavioral tasks. In the automated video analysis of spontaneous physical activity (SPA), the same mice were analyzed repeatedly at 3, 5, 8, and 10 months of age (Figure 1A). The mice analyzed in the SPA were not used for any of the other behavioral tasks. Figure 1B indicates the procedures of the six learning paradigms. In both the reference and reverse memory paradigms of the MWM, mice were only used in one of the two paradigms. A separate set of mice were used in the novel object recognition test and fear conditioning experiments. The same mice were first used for the novel object recognition task, followed 1 week later by the fear conditioning experiment, to limit the potential impact of the first task on the second one. Similarly, another set of mice were prepared and they were used for the water-finding test and trace eyeblink conditioning, with a 1-week interval in between the two tests. The age of the mice during the six learning paradigms fell within 3–5 months of age.

#### Automated Video Analysis of Spontaneous Physical Activity in the Home Cage

**Figure 2:**
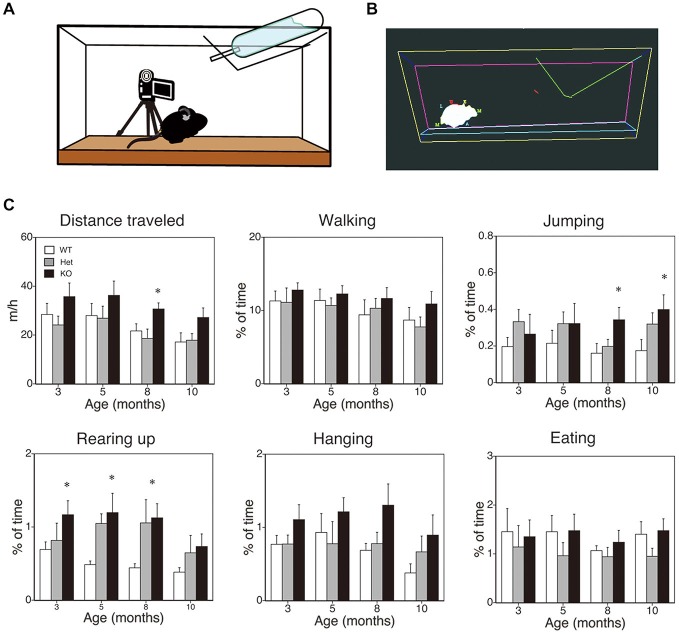
**SPA in mice deficient in monoacylglycerol lipase (MGL) knockout (KO) mice**. SPA in MGL KO mice. SPA was evaluated in the control (MGL^+/+^; *n* = 10), Het (MGL^+/−^; *n* = 10), and MGL KO (MGL^−/−^; *n* = 10) mice at the ages of 3, 5, 8, and 10 months. **(A)** Schematic diagram for evaluating spontaneous behavioral changes by an automated video-based behavior analysis system (HomeCageScan). The mouse behaviors were recorded by video cameras mounted on tripods that were angled perpendicularly to the cage, to provide a side view of the cage. **(B)** An example of mouse behavior analyzed by the HomeCageScan system. The yellow, pink, and cyan lines indicate the edges of the cage. The green line depicts the feeding basket. The gray shape indicates the mouse’s body. **(C)** Six separate parameters of spontaneous behavior, i.e., distance traveled, walking, jumping, rearing, hanging, and eating, were evaluated for 3 h in the home cage. MGL KO mice exhibited significantly higher SPA, with regard to distance traveled, rearing, and jumping, at several stages. *p < 0.05 relative to the corresponding control group.

The method employed here was essentially the same as that described previously (Chen et al., 2005; Pfluger et al., 2011; Hiasa et al., 2013; Kishimoto et al., 2013). A schematic diagram is shown in Figure 2A. Mice that were 3, 5, 8, or 10 months of age were transferred to a new home cage (21 × 31 × 12 cm), and were video recorded for 3 h from 10:00 to 13:00. A camcorder (NV-GS300, Panasonic Corporation, Tokyo, Japan) was mounted on a tripod that was angled perpendicular to the cage to provide a side view of the cage. The camera footage was transferred to and saved on a Dell computer with the mAgicTV software (I-ODATA DEVICE, Inc., Kanazawa, Japan). The video data were analyzed using the CleverSys HomeCageScan system (CleverSys Inc., Reston, VA). Spontaneous behaviors such as locomotor activity, rearing, and hanging were evaluated.

#### MWM

The procedures of acquisition and extinction in the MWM were performed as previously described (Varvel and Lichtman, 2002; Varvel et al., 2006, 2007; Kishimoto et al., 2013). The water maze pool (Eiko Science, Tokushima, Japan), which had a diameter of 120 cm, contained opaque, white water (22 ± 2°C) with a translucent platform (10 cm in diameter) that was submerged 1 cm below the surface. Four sheets of paper with black and white geometric designs were attached to the walls of the experimental room as additional cues. If the mice could not reach the platform, the latency was set at 80 s. In the acquisition phase of reference memory, the platform location remained constant, and the entry points were changed semi-randomly between trials. Before the acquisition phase, each animal performed a single 5-min acclimation trial (Varvel et al., 2006). The acquisition phase of the hidden platform task took 10 days to perform (2 trials per day). One day after the acquisition phase, a 2-min extinction phase (2 trials/day, 5 days) was performed without the platform. The entry point for the extinction trial was the quadrant opposite to the target quadrant. In the extinction trials, the escape latency was defined as the time required to find the location where the platform used to be during the acquisition phase. The time that mouse spent in the each quadrant was also evaluated during the extinction trials. In the reversal learning paradigm, during the first 10 trials (acquisition phase), the hidden platform was held in the same position; the platform was then moved to the opposite quadrant for the remaining 5 trials (reversal phase). About 24 h after the completion of the reversal phase, mice were subjected to a 2-min probe trial (Tsetsenis et al., 2011). Performance was monitored and analyzed with an automated video tracking system (CleverSys Inc., Reston, VA).

#### Novel Object Recognition Task

The task was conducted in open-field chambers (25 cm × 25 cm) made of gray Plexiglas (O’Hara and Co., Tokyo, Japan). Mice (*n* = 10 per genotype) were first habituated to the chamber for 20 min per day for 3 days. On the third day, two identical objects were placed in the open field to acclimatize the mice to the test conditions. The following day, during training, mice were allowed to explore two new objects with different shapes for 10 min. One hour later, mice were reintroduced into the open field for 5 min with two objects, a copy of a familiar object and a new object (1-h test). The next day, mice were again allowed to explore two objects for 5 min (24-h test), including a copy of the second familiar object and a new object. Behavioral activity during training and testing was videotaped and analyzed by observers blind to the genotype. A mouse was considered as exploring an object when its head was oriented directly toward the object and within 2 cm from it. Posturing and mounting were not included in the analysis. Mice have a natural tendency to explore novel objects compared with familiar objects. To compare the effects of MGL deletion with the effects of CB1 receptor deletion, we used the recognition index calculated by dividing the amount of time spent on the novel object by the total time spent on both objects; this index was used previously to evaluate recognition memory in CB1 KO mice (Reibaud et al., 1999). Two MGL KO mice and one Het mouse were excluded from statistical analysis, since they did not explore the objects during the 1-h test.

#### Contextual Fear Conditioning

Mice were placed into the conditioning chamber (310 × 250 × 280 mm; O’Hara and Co., Tokyo, Japan) for 3 min before the onset of the unconditioned stimulus (US) (footshock; 2 s, 0.2 mA) to allow them to explore the testing chamber and develop a representation of the context. After the footshock, the mice were left in the chamber for an additional 30 s before returning to their home cage. To evaluate the extinction of contextual fear conditioning, the mice were placed back in the conditioning chamber for 3 min per day, for four consecutive days, starting 24 h after conditioning. Mice were videotaped and their freezing behavior (absence of all movement except respiration) was measured by observers blind to the genotype and expressed as the percentage of freezing.

#### Trace Eyeblink Conditioning

The surgery and conditioning procedures were performed as previously described (Kishimoto and Kano, 2006; Kishimoto et al., 2006). Mice were anesthetized with ketamine (80 mg/kg, i.p.; Sankyo, Tokyo, Japan) and xylazine (20 mg/kg, i.p.; Bayer, Tokyo, Japan), and four Teflon-coated stainless steel wires (100 μm in diameter; A-M Systems, WA) were subcutaneously implanted under the left eyelid. Two of the wires were used to deliver the US, and the remaining two were used to obtain an electromyogram (EMG) from the musculus orbicularis oculi, which is responsible for eyelid closure. A 352-ms tone (1 kHz, 80 dB) was used as the CS, and a 100-ms electrical shock (0.2 mA, 100 Hz square pulses) was used as the US. Each session consisted of 100 trials, grouped in 10 blocks. An individual session consisted of 10 CS alone (every tenth trial), and 90 paired CS-US trials. The intertrial interval was randomized between 20 and 40 s, with a mean of 30 s. The spontaneous eyeblink frequency was measured by 100 “no-stimulus” trials during acclimation, 1 day before the conditioning experiment began. The CS and US were separated by a stimulus-free interval (trace interval of 500 ms). Mice received 10 days of acquisition and a subsequent 4 days of extinction sessions. By monitoring the EMG and body responses of the mice in the first several CS-US trials, we carefully determined the US intensity as the minimal current amplitude required to elicit an eyeblink response (conditioned response, CR) and a constant unconditioned response (UR). The US intensity was adjusted daily for each animal. CR and UR amplitudes were defined as the EMG amplitude at the time 50 ms before or after the US, respectively. The CR peak was defined as the latency from CS onset of the maximum eyelid EMG within 852 ms from CS onset. All experiments, including the surgery, were performed by an operator who was blind to the genotype of the mice. The EMGs were analyzed as described previously (Kishimoto and Kano, 2006; Kishimoto et al., 2006). A threshold was determined, and the time window selected for evaluating the CR was 200 ms before US onset.

#### Water-Finding Test

The apparatus and experimental procedure were essentially the same as those as described previously for evaluating latent visuospatial learning (Ettenberg et al., 1983; Ichihara et al., 1993; Kishimoto et al., 2013). In this study, we applied the video tracking system, TopScan (CleverSys Inc., Reston, VA) in the conventional latent learning apparatus. The apparatus consisted of an open field (40 × 72 × 30 cm), with an alcove (15 × 20 × 10 cm) in the middle of one of the long walls. A metal drinking tube, of the same type as that in the home cages, was inserted in the center of the alcove ceiling at 5 cm (in the training trials) or 7 cm (in the test trials) above the floor. In the training trials, the water pot was left empty, and the animals were not previously deprived of water. Each mouse was placed individually in the starting corner, and the time that elapsed before the mouse began to explore the environment was recorded as the starting latency (SL). The mouse was then allowed to explore the environment freely for 3 min. If a mouse could not touch the drinking tube, the mouse was excluded from subsequent test trials (2 mice were excluded). After the training trials, the mice were quickly returned to their cages and were deprived of water until the test trial. On the next day, the test trials were conducted in the apparatus. The period from the onset of exploration to entering the small alcove was defined as the entering latency (EL). The period from first entering the alcove to touching the drinking tube was defined as the finding latency (FL). The sum of the EL and FL was designated as the drinking latency (DL). If the mice failed to find the drinking tube during the 3-min period from the onset of exploration, DLs were recorded as 180 s. The test trials were conducted as the training trials were.

### Statistical Analysis

Data obtained in all behavioral tests were analyzed using the one-way or repeated-measures ANOVA, followed by a *post hoc* Scheffe’s test, using the SPSS program (IBM Corporation, Armonk, NY). All data are presented as the mean ± the standard error of the mean (SEM). Significance was assigned at *p* < 0.05.

## Results

### Age-Related Changes in the Spontaneous Behaviors of MGL KO Mice

First, to evaluate the role of 2-AG in spontaneous physical activities, such as voluntary running, we cross-sectionally analyzed the voluntary behaviors of MGL KO mice in the home-cage environment (Figures 2A,B). Six separate behaviors were identified and evaluated for animal movement, i.e., distance traveled, walking, jumping, rearing up, hanging, and eating (Figure 2C), using the HomeCageScan software (Chen et al., 2005; Pfluger et al., 2011). MGL KO mice exhibited increased activity with regard to some spontaneous behaviors, in an age-dependent manner. Although a significant difference in the distance traveled was observed between control (WT and Het mice) and MGL KO mice only at 8 months of age (Figure 2C; *F*_(2,27)_ = 5.10, *p* = 0.013; *Post hoc* tests: *p* < 0.05, for both WT vs. KO and Het vs. KO), there was a tendency of increased distance traveled in MGL KO mice from the age of 3 months. On the other hand, significant statistical differences were observed in rearing up behavior between the WT and MGL KO mice from the age of 3 to 8 months (Figure 2C; 3 months: *F*_(2,27)_ = 3.57, *p* = 0.042; *Post hoc* tests: *p* < 0.05, for WT vs. KO at the 3 months, and *p* < 0.05, for both WT vs. KO and Het vs. KO at the ages of 5 and 8 months). The frequency of jumping behavior increased in MGL KO mice from the age of 8 months onward (Figure 2C; 8 months: *F*_(2,27)_ = 3.35, *p* = 0.042). In contrast, walking, hanging, and eating behaviors did not appear affected at any age (Figure 2C). We concluded that motor activities were largely normal in MGL KO mice at 3–5 months of age, although several voluntary behaviors, especially exploratory behaviors, such as rearing-up, were affected at that age. Hence, MGL KO mice at the age of 3–5 months were subjected to the following behavioral learning and memory tests.

### Enhanced Extinction and Reversal Learning of the MWM in MGL KO Mice

**Figure 3:**
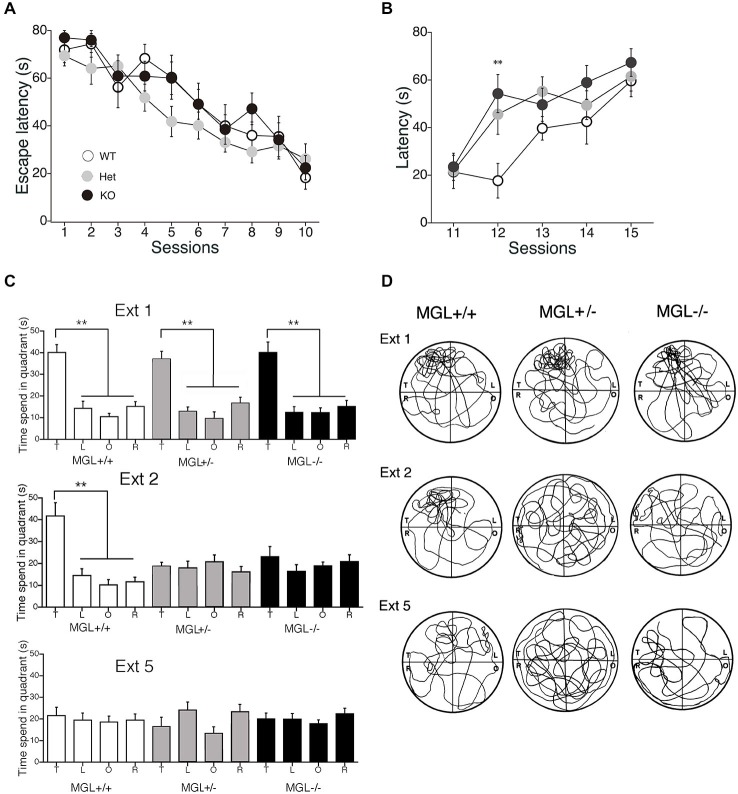
**Accelerated extinction of reference memory in the MWM in MGL KO mice. (A)** WT (MGL^+/+^; *n* = 10), Het (MGL^+/−^; *n* = 10), and MGL KO (MGL^−/−^; *n* = 15) mice were trained in the water maze paradigm for 10 consecutive days (2 trials per day). The platform was fixed in a target quadrant. Escape latencies for both groups improved throughout training, and there was no significant difference between the three groups. **(B)** After the acquisition phase, the platform was removed, and the extinction of reference memory was tested in WT (MGL^+/+^; *n* = 10), Het (MGL^+/−^; *n* = 10), and KO (MGL^−/−^; *n* = 15) mice. Escape latencies of MGL KO and Het mice were significantly longer compared to those of WT mice. *Post hoc* tests: *p* < 0.01 at the 12th session, for both WT vs. Het and WT vs. KO. **(C)** Learning performance at the extinction phase is expressed as the percentage of time spent in each of the water-maze quadrants (T: target quadrant; L: left quadrant; O: opposite quadrant; R: right quadrant). In the first session of the extinction phase (Ext 1), all three groups spent significantly more time in the target quadrant. In the second session of the extinction phase (Ext 2), WT mice still spent significantly more time in the target quadrant. In contrast, MGL KO mice no longer showed a preference for the target quadrant. **(D)** Representative images of the swimming paths of mice during the extinction sessions. **p < 0.01 relative to the corresponding control group.

**Figure 4:**
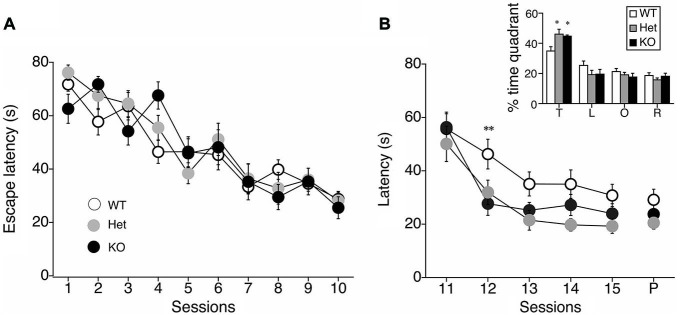
**Facilitation of reversal learning in the MWM in MGL KO mice. (A)** The latencies for reaching the hidden platform during the acquisition phase of the MWM task for control (MGL^+/+^; *n* = 14), Het (MGL^+/−^; *n* = 12), and MGL KO (MGL^−/−^; *n* = 15) mice. Mice were trained in the water maze paradigm for 10 consecutive acquisition sessions (2 trials per day). The platform was located in the target quadrant. No differences in latency were observed between genotypes. **(B)** After the acquisition phase, the platform was moved to the opposite side of the tank, and the reversal of spatial memory was tested in MGL KO mice. Escape latencies of MGL Het and KO mice were significantly lower compared to WT mice. MGL KO mice showed faster reversal learning than their control littermates. B (inset), Percentage of time spent in each quadrant (T: target quadrant; L: left quadrant; O: opposite quadrant; R: right quadrant) during a single probe trial following reversal training. MGL Het and KO mice spent more time in the new target quadrant, suggesting that they were able to reverse their learning of the platform location. **p < 0.01; *p < 0.05 compared with their corresponding control group.

We next performed a battery of behavioral assays to determine whether hippocampus-dependent spatial learning was altered in MGL KO and Het mice (Figure 3). First, MGL KO mice and their WT and Het littermates were trained in the MWM paradigm for 10 consecutive days. In the acquisition phase, the platform was fixed in a target quadrant, and escape latencies improved for both groups throughout training. No significant differences were observed between the 3 groups (*F*_(2,32)_ = 1.996, *p* = 0.154 for the genotypic effect; *F*_(18,288)_ = 0.719, *p* = 0.791 for the genotype × session interaction; Figure 3A). After the acquisition phase, the platform was removed, and the extinction of reference memory was tested in WT, Het, and MGL KO mice. The escape latencies of MGL KO and Het mice were significantly longer than that of control mice (*F*_(2,32)_ = 3.345, *p* = 0.048 for the genotypic effect; *F*_(8,128)_ = 1.453, *p* = 0.181 for the genotype × session interaction), indicating that MGL KO and Het mice exhibited rapid extinction of reference memory (Figure 3B). Figure 3D depicts representative images of the swimming paths of mice during the extinction sessions, and indicates a rapid extinction of spatial memory. In Ext 2, control mice still exhibited a preference for the original target quadrant, but MGL KO mice no longer exhibited the preference. Figure 3C indicates the learning performance at the extinction phase expressed as a percentage of the time spent in each of the water-maze quadrants. In the first session of the extinction phase (Ext 1), WT, Het, and MGL KO mice spent significantly more time in the target quadrant (WT: *F*_(3,36)_ = 23.585, *p* < 0.01; Het: *F*_(3,36)_ = 19.813, *p* < 0.01; KO: *F*_(3,56)_ = 16.824, *p* < 0.01). In the second session of the extinction phase (Ext 2), control mice still spent significantly more time in the target quadrant (*F*_(3,36)_ = 15.873, *p* = 0.00000097). In contrast, MGL Het and KO mice no longer showed a preference for the target quadrant (*F*_(3,36)_ = 0.623, *p* = 0.605 for Het mice; *F*_(3,56)_ = 0.746, *p* = 0.529 for KO mice). In the last session of the extinction phase (Ext 5), the WT mice also had a disrupted memory for the target quadrant preference. Thus, there was no difference in the time spent in the target quadrant between the control and KO mice. We then evaluated the reversal learning of the MWM task in MGL KO mice (Figure 4). The acquisition of reference memory was normal (Figure 4A; *F*_(2,39)_ = 0.761, *p* = 0.474 for the genotypic effect; *F*_(8,312)_ = 0.795, *p* = 0.607 for the genotype × session interaction). However, when the position of the platform was reversed, MGL KO and Het mice showed significantly shorter escape latencies than their control littermates (*F*_(2,41)_ = 9.695, *p* = 0.00036 for the genotypic effect; *F*_(8,164)_ = 0.672, *p* = 0.715 for the genotype × session interaction; Figure 4B). *Post hoc* comparisons indicated significant differences between WT and Het mice and between WT and KO mice (*p* = 0.019 and 0.028, respectively). At a single probe trial following reversal training, MGL KO mice displayed a strong preference for the new target quadrant (*F*_(2,41)_ = 4.972, *p* = 0.012; the inset of Figure 4B). *Post hoc* tests revealed significant differences between WT and Het mice and between WT and KO mice (*p* = 0.034 and 0.045, respectively). These data suggest that although MGL deletion does not alter spatial learning capabilities, it seems to facilitate the reversal of spatial learning in mice.

### Increased Memory Retention in the Object Recognition Task in MGL KO Mice

**Figure 5:**
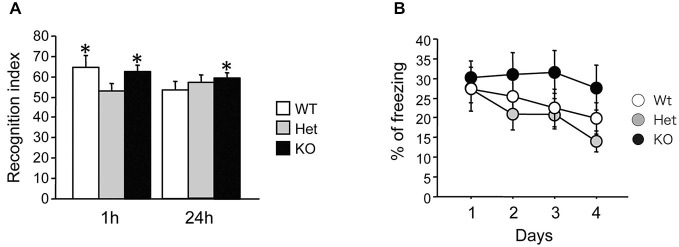
**Increased recognition memory retention in MGL KO mice, but normal contextual fear memory in MGL Het and KO mice. (A)** Novel object recognition was tested in control (MGL^+/+^; *n* = 10), Het (MGL^+/−^; *n* = 9), and MGL KO (MGL^−/−^; *n* = 8) mice. The recognition index for the novel object recognition test is shown. MGL KO mice still showed a recognition index above chance level when tested 24 h later. Het mice did not recognize the familiar object either 1-h or 24-h after acquisition. *p < 0.05 compared to 50%. **(B)** Contextual fear conditioning was tested in control (MGL^+/+^; *n* = 10), Het (MGL^+/−^; *n* = 10), and MGL KO (MGL^−/−^; *n* = 10) mice. The percentage of freezing during the four daily sessions of contextual fear conditioning testing is shown. There was no difference between the genotypes.

We then tested novel object recognition in MGL KO mice. The recognition indexes of the 1-h and 24-h tests are displayed in Figure 5A. During the 10-min training session, mice showed similar interactions with the objects (data not shown; WT mice: 25.6 ± 5.1, Het mice: 31.3 ± 4.2, KO mice: 41.7 ± 7.6; ANOVA: *F*_(2,24)_ = 2.06, *p* = 0.15). During testing, if the mice remembered the familiar object, they naturally spent more time exploring the novel object. When tested 1 h after training, both WT and KO mice, but not Het mice, explored the novel object more than the familiar one (Student’s *t*-test compared to 50%; WT mice: *t*_(9)_ = 2.51, *p* = 0.03; Het mice: *t*_(8)_ = 0.7, *p* = 0.5; KO mice: *t*_(7)_ = 3.57, *p* = 0.009). However, when tested 24 h later, only KO mice showed a recognition index that was significantly different from 50%, indicating spared memory of the familiar object (WT mice: *t*_(9)_ = 0.84, *p* = 0.42; Het mice: *t*_(8)_ = 2.01, *p* = 0.08; KO mice: *t*_(7)_ = 3.75, *p* = 0.007). Taken together, these data show that long-term recognition memory retention was enhanced in MGL KO mice.

### Contextual Fear Conditioning in MGL KO Mice

MGL KO mice were subjected to contextual fear conditioning, which involved placing the animals in an environment where they received electric footshocks. MGL KO mice did not differ from control and Het mice in their basal freezing behavior (3 min habituation before the onset of the shock) or post-shock freezing behavior on the conditioning day (data not shown; basal: *F*_(2,27)_ = 0.86, *p* = 0.43; post-shock: *F*_(2,27)_ = 1.33, *p* = 0.28). As shown in Figure 5B, although KO mice tended to have a slower extinction of contextual fear conditioning, there was no difference between the genotypes in the percentage of freezing during extinction (genotype: *F*_(2,27)_ = 1.34, *p* = 0.28; days: *F*_(3,81)_ = 4.94, *p* = 0.003; interaction: genotype × days: *F*_(6,81)_ = 0.95, *p* = 0.46). Thus, extinction of conditioned fear appeared to be slightly impaired in MGL KO mice, when compared with their WT and Het littermates.

### Normal Acquisition and Extinction of Non-Spatial Trace Eyeblink Conditioning in MGL KO Mice

**Figure 6:**
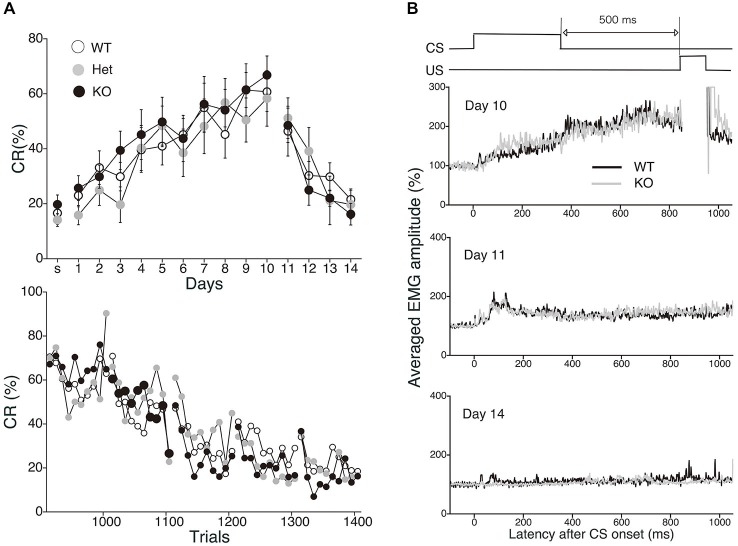
**Normal acquisition and extinction of trace eyeblink conditioning in MGL KO mice. (A)** Long-trace interval (TI) eyeblink conditioning (trace interval (TI) = 500 ms) was investigated in MGL KO (open circle; *n* = 10), Het (gray circle; *n* = 10), and WT mice (closed circle; *n* = 10), which were used as control mice. The daily CR% (upper) and the block CR% of 10 trials (lower) are plotted. **(B)** Schematic representation of the trace eyeblink conditioning paradigms (top panel). Averaged EMG amplitudes were compared between control (MGL^+/+^; *n* = 10) and MGL KO (MGL^−/−^; *n* = 10) mice on day 10, 11, and 14 (lower panel).

To evaluate the physiological role of 2-AG in non-spatial hippocampal learning, we tested the acquisition and extinction of trace eyeblink conditioning with a 500-ms trace interval (TI) in MGL KO mice (Figure 6). MGL KO mice, Het mice, and control mice successfully acquired the CRs during the 10 daily sessions, and the level of CR attained was about 60% on day 10 (Figure 6A). There was no difference in acquisition performance between the 3 groups. ANOVA revealed no significant interaction effects between sessions or groups (*F*_(18,243)_ = 0.351, *p* = 0.99) and no significant group effect (*F*_(2,27)_ = 1.66, *p* = 0.21). In the extinction session, MGL KO mice and control mice successfully acquired CRs during the 4 days (days 11–14). ANOVA revealed no significant interaction effect between the sessions or groups (*F*_(6,81)_ = 0.533, *p* = 0.782) and no significant group effect (*F*_(2,27)_ = 0.584, *p* = 0.565). There was also no difference between the groups in the intraday acquisition and extinction of CRs (lower panel of Figure 6A). Figure 6B shows the averaged EMG amplitudes of control and MGL KO mice on days 10, 11, and 14. The amplitude and temporal pattern of eyelid EMG of MGL KO mice were not altered relative to those of control mice on any day. CR amplitude, UR amplitude, and CR peak latency were also unchanged in MGL KO mice (Table 1). Taken together, these findings indicate that the long-trace eyeblink conditioning was normal in MGL KO mice.

**Table 1:** **CR amplitude, UR amplitude, and CR timing during trace eyeblink conditioning in MGL KO mice**.

		WT		KO	
		Average	SEM	Average	SEM	*t*-test vs. WT
Day 10	CR amplitude (%)	218.6	10.9	212.4	7.4	0.88
	UR amplitude (%)	356.9	26.9	326.7	12.1	0.79
	CR peak latency (ms)	509.7	51.1	564.7	41.3	0.71
Day 11	CR amplitude (%)	142.5	2.9	144.5	4.6	0.91
	CR peak latency (ms)	489.4	26.9	548.9	29.8	0.178
Day 14	CR amplitude (%)	118.1	2.2	110.7	1.7	0.14
	CR peak latency (ms)	614.8	53.7	559.6	55.8	0.49

*The amplitudes of CR and UR, and the peak latency of CR were calculated during long-trace interval eyeblink conditioning in MGL KO mice. Values are group mean ± SEM*.

### Enhanced Visuospatial Latent Memory in MGL KO Mice

**Figure 7:**
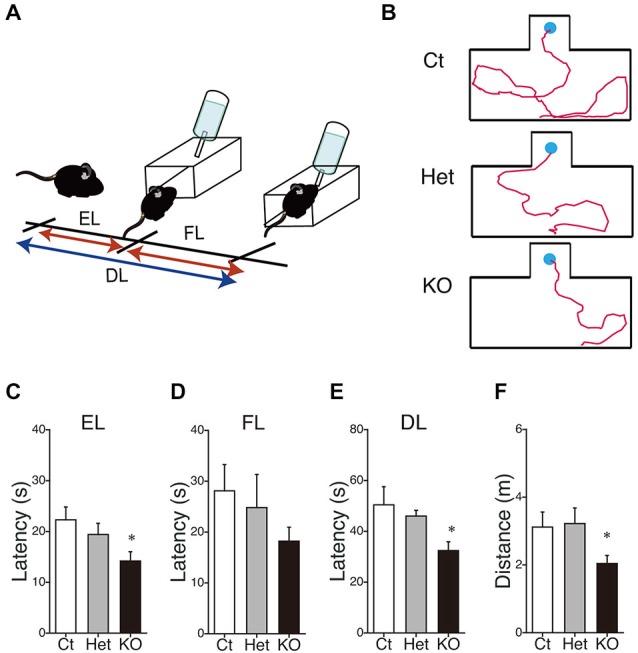
**Enhancement of latent visuospatial memory in MGL KO mice. (A)** Schematic representations of the entering latency (EL) and finding latency (FL). The period from the onset of exploration to entering the small alcove was defined as the EL, and the period from entering the alcove until finding the drinking tube was defined as the FL. The sum of the EL and FL was designated as the drinking latency (DL). **(B)** Typical images depicting the walking paths during the test trials in control (upper), Het (middle), and MGL KO mice (lower). **(C–F)** The EL **(C)**, FL **(D)**, and DL **(E)** for the water-finding test were evaluated in control (open bar, *n* = 10), Het (gray circle; *n* = 10), and MGL KO mice (closed bar, *n* = 10). The distance traveled **(F)** from the starting point to the drinking tube was also evaluated in MGL KO mice. The EL, DL, and distance traveled by MGL KO mice were significantly decreased relative to those of control or Het mice. *p < 0.05 relative to the control group.

Finally, we performed the water-finding test for evaluating visuospatial latent learning in MGL KO mice (Figure 7). In the present study, for quantitative analysis of the latencies and distances, we used an automated tracking analysis system (TopScan, CleverSys) equipped with a video camera from above. Figure 7A shows schematic representations of the EL and FL. The period from the onset of exploration to entering the small alcove was defined as the EL, and the period from entering the alcove until finding the drinking tube was defined as the FL (Figure 7A). Figure 7B depicts representative images of the test trial walking paths for control and MGL KO mice. MGL KO mice entered the small alcove comparatively quickly, and walked a short distance before finding the drinking tube. Quantitative analysis indicated that the ELs of MGL KO mice were significantly shorter than those of control and Het mice (Figure 7C; *F*_(2,27)_ = 3.66, *p* = 0.039; *Post hoc* tests: *p* < 0.05, for WT vs. KO, but no differences between Het and KO), although there was no significant difference in FL (Figure 7D; *F*_(2,27)_ = 0.99, *p* = 0.38). The DLs of MGL KO mice were slightly shorter than those of control and Het mice (Figure 7E; *F*_(2,27)_ = 1.99, *p* = 0.16; *Post hoc* tests: *p* < 0.05, for WT vs. KO, but no differences between Het and KO). The distances traveled before finding the drinking tube were also decreased in MGL KO mice (Figure 7F; *F*_(2,27)_ = 3.89, *p* = 0.033). The SL, EL, and distance traveled during the training trial were not altered in MGL KO mice (Table 2).

**Table 2:** **Starting latency, entering latency, and the distance traveled by the MGL KO mice in the training trials of water-finding test**.

	SL (s)		EL (s)		distance traveled (m)	
	WT	KO	WT	KO	WT	KO
	7.4 ± 1.2	5.0 ± 1.6	35.8 ± 4.9	22.4 ± 5.3	3.0 ± 0.3	3.7 ± 0.5
*t*-test vs. WT	0.19		0.08		0.26	

*The starting latency (SL), entering latency (EL), and distance traveled from the starting point to touching the water bottle during the training trials of the water-finding test were evaluated in the MGL KO mice at 3 months of age. Values are the group mean ± SEM*.

## Discussion

The current study investigated the functional role of 2-AG in SPA and hippocampus-dependent learning by using MGL Het and KO mice. Our results clearly show higher SPA in aged MGL KO mice, and altered performance in several learning tasks in MGL Het and/or MGL KO mice. The most abundant endocannabinoid, 2-AG, plays a critical role in cannabinoid receptor-mediated cell signaling (Sugiura et al., 1997, 1999; Sugiura and Waku, 2000; Kano et al., 2009; Sugiura, 2009). It also has important functions in degenerative and inflammatory disorders of the CNS (Awumey et al., 2008; Woodward et al., 2008; Bisogno and Di Marzo, 2010; Fowler, 2012; Mulvihill and Nomura, 2013; Valdeolivas et al., 2013). Despite the physiological importance of 2-AG, its role in cognitive function remains poorly understood, though a few attempts have been made towards elucidating its behavioral roles (Long et al., 2009; Pan et al., 2011; Griebel et al., 2015). We have recently reported that 2-AG signaling negatively regulates habituation (Sugaya et al., 2013). In contrast, many reports are available on the behavioral effects of AEA, using FAAH KO mice or pharmacological FAAH inhibition (Varvel et al., 2006, 2007; Moreira et al., 2008; Mazzola et al., 2009; Wise et al., 2009; Bambico et al., 2010; Cassano et al., 2011). The generation of MGL KO mice allows us to evaluate hypotheses regarding the essential role of MGL in behavioral function. In the present study, a battery of cognitive tests was administered to MGL KO mice, and the results were compared to those from their WT and Het littermates. We found that MGL KO mice exhibited behavioral and cognitive alterations on several tasks, particularly enhanced extinction and reversal learning in the MWM and a tendency for slower extinction in contextual fear conditioning.

### Increased Exploratory Behavior and Motor Activities in MGL KO Mice

We first showed that spontaneous activity, especially rearing up, was enhanced in MGL KO mice from the young adult stage (Figure 2C). These results indicate that exploratory behavior and locomotor activity were increased by the deletion of MGL. Accumulating data suggest that CB_1_ signaling plays an important role in motor and voluntary behaviors (El Manira and Kyriakatos, 2010), and CB_1_ KO mice show increased thigmotaxis in an open field, decreased distance traveled, and decreased wheel-running activity (Jacob et al., 2009; Dubreucq et al., 2010). Our results of enhanced rearing were also consistent with the findings of a previous study showing that endocannabinoids facilitate exploratory behavior (Jacob et al., 2009). We conclude that 2-AG is at least partially responsible for these motor and spontaneous behavioral alterations observed in CB_1_ KO mice.

### Enhanced Extinction and Reversal Learning of Hippocampus-Dependent Spatial Memory

Many studies have reported that spatial memory performance is impaired in chronic marijuana users, suggesting an important role of the CB_1_ receptor in hippocampal memory (Kanayama et al., 2004; Padula et al., 2007; Sneider et al., 2013). Furthermore, using an animal model, the roles of the CB_1_ receptor in hippocampal plasticity and learning have been intensively investigated in the last decade (Stella et al., 1997; Carlson et al., 2002; Mereu et al., 2003; Chevaleyre and Castillo, 2004; Varvel et al., 2005). CB_1_ blockade induces enhanced hippocampal-CA1 long-term potentiation (LTP), impaired extinction of reference memory, and impaired reversal learning in the MWM, which is a commonly used apparatus for measuring visuospatial reference memory in rodents (Carlson et al., 2002; Varvel et al., 2005). Varvel et al. discovered accelerated acquisition and extinction rates in the MWM, and concluded that endogenous anandamide facilitates extinction through a CB_1_ receptor mechanism (Varvel and Lichtman, 2002; Varvel et al., 2007). In the present study, we clearly show that MGL KO mice exhibited selective enhancement of extinction learning during the MWM task, suggesting that 2-AG also plays a critical role in the extinction process of spatial memories (Figure 3). Thus, our present findings supported the notion that 2-AG is at least partially responsible for facilitating the extinction and/or forgetting processes of spatial memory. On the other hand, MGL KO mice also showed enhanced acquisition of reverse memory in the MWM when the position of the platform was reversed (Figure 4). However, the enhanced reverse memory might be reflected in the accelerated extinction of learned information in the mutant lines. Indeed, the 12th session of reversal training, in which both MGL KO and Het mice exhibited lower escape latencies (Figure 3B), corresponds well to the second session of the extinction phase (Figure 4B). In fact, we cannot strictly distinguish the extinction process from the reverse memory formation process by the present experiment. It is possible that we observed essentially the same effects of MGL deletion on cognitive function in the two MWM paradigms. If we interpret the extinction results from the other viewpoint, Het and KO mice rapidly learned that the platform was no longer in the original location. Additionally, in the reversal learning paradigm, Het and KO mice rapidly learned that the platform had been moved to a new location. Thus, our MWM results might indicate that Het and KO mice can quickly re-learn new spatial information by responding to an environmental change without adhering to old information. This idea is consistent with several previous reports suggesting the importance of the endocannabinoid system in behavioral and cognitive flexibility (Hill et al., 2006; Robinson et al., 2008; Klugmann et al., 2011).

Spatial learning in the MWM is thought to be critically dependent on postsynaptic, NMDA receptor-dependent LTP of synaptic transmission; however, the contribution of presynaptic forms of long-term plasticity to learning and memory remains unclear. Moreover, the relationship between hippocampal plasticity and extinction learning is also poorly understood. These issues should be addressed in future experiments on animal models.

### Enhanced Recognition Memory Retention and Slower Extinction of Contextual Fear Memory in MGL KO Mice

MGL KO mice displayed increased memory retention in the object recognition task, as they had a recognition index significantly above 50% when tested 24 h later (Figure 5A). This result suggests that extinction of recognition learning is impaired in MGL KO mice. This behavioral phenotype is similar to that of CB_1_ KO mice (Reibaud et al., 1999; Maccarrone et al., 2002). On the other hand, extinction of contextual fear conditioning also tended to be slower in MGL KO mice (Figure 5B). In the CB_1_ KO mice, extinction of tone fear conditioning is disrupted (Marsicano et al., 2002; Kamprath et al., 2006), and pharmacological approaches indicate a facilitatory role of the endocannabinoid system in fear memory extinction (Pamplona et al., 2006; Bitencourt et al., 2008; Campolongo et al., 2009; Das et al., 2013; Laricchiuta et al., 2013). Thus, our current results are apparently contradictory. Taking into account the fact that MGL Het mice, unlike MGL KO mice, showed enhanced extinction of novel object recognition memory or reduced memory consolidation (Figure 5A), long-term endocannabinoid compensatory changes (CB_1_ downregulation) might be induced in MGL KO mice (Schlosburg et al., 2010). It is likely that due to this compensatory effect, MGL KO mice act similar to CB_1_ KO mice in the extinction phase of contextual fear conditioning.

### Normal Acquisition and Extinction of Hippocampus-Dependent Trace Eyeblink Conditioning

Eyeblink conditioning can mainly be classified into two distinct types: delay and trace conditioning paradigms (Thompson and Krupa, 1994). Trace conditioning with a sufficiently long stimulus-free interval depends on the hippocampus in several species (Woodruff-Pak, 2000). We previously demonstrated that long-interval trace eyeblink conditioning also requires an intact hippocampus in WT mice (Kishimoto et al., 2001a, 2006), and that this conditioning is more susceptible to normal age- and Alzheimer’s disease-related memory deterioration than delay conditioning in mice (Kishimoto et al., 2001b, 2012). However, our present findings indicate that, unlike the MWM, trace eyeblink conditioning was normal in MGL KO mice at both the acquisition and extinction phases (Figure 6). The results are in agreement with our previous study showing that trace eyeblink conditioning is normal in CB_1_ KO and CB_1_ antagonist-injected mice, although memory formation of cerebellum-dependent delay eyeblink conditioning is impaired in CB_1_ KO mice (Kishimoto and Kano, 2006). In human subjects, heavy cannabis users exhibit normal conditioned responses in trace eyeblink conditioning relative to controls in the acquisition and extinction phases, although the cannabis group exhibits severe impairment of delay eyeblink conditioning acquisition (Edwards and Skosnik, 2007; Edwards et al., 2008; Skosnik et al., 2008). Taken together, the task-specific dependency of 2-AG signaling is consistent with that of CB_1_ signaling. Our results do not contradict the hypothesis that the CB_1_ receptor plays a vital role in the extinction of aversive memories, but is not essential for the extinction of learned responses, in appetite-motivated tasks (Niyuhire et al., 2007). Our present results extend this concept by suggesting that the CB_1_ receptor is not essential for the extinction of non-motivational learning.

### Enhanced Visuospatial Latent Memory in MGL KO Mice

The water-finding test is based on a latent visuospatial memory and learning paradigm related to attentional processes and the ability to sort visuospatial information (Ettenberg et al., 1983; Ichihara et al., 1993). This learning paradigm does not require motivation nor does it involve compulsion, and the animals can freely explore the environment (Ichihara et al., 1993; Mouri et al., 2007). The molecular mechanisms and brain regions critical for the water-finding test are not fully understood, but dopaminergic and NMDA-ergic processes were suggested to be involved in this type of learning (Ichihara et al., 1993; Miyamoto et al., 2001; Mouri et al., 2007). Moreover, increased hippocampal LTP and enhanced performance on the water-finding test were observed in a line of mutant mice, implying critical hippocampal dependency for this type of learning (Nakamura et al., 2001). Our results showing cognitive enhancement in MGL KO mice support the hypothesis that CB_1_ signaling, mediated by 2-AG, is important for cognitive functioning, although it may also be due to enhanced exploratory behavior or attention (Figure 2). Indeed, even in the training trial (day 1), MGL KO mice tended to enter the small alcove rapidly compared to control mice, though this difference was not statistically significant (Table 2). In fact, growing evidence suggests the involvement of the endocannabinoid system for modulating attention and exploratory behavior (Jacob et al., 2009; Castelli et al., 2011; Häring et al., 2011; Ford et al., 2014).

## Conclusions

In summary, our behavioral study demonstrates that MGL KO mice exhibited task-specific enhancement of extinction in hippocampus-dependent learning, suggesting a vital role for 2-AG signaling in extinction and reversal learning. Recently, Pan et al. (2011) reported that mutant mice deficient in MGL exhibit enhanced acquisition of hippocampus-dependent learning, including reference memory in the MWM. Our results showing normal acquisition and enhanced extinction of hippocampus-dependent spatial learning contradict this recent report, but could be explained by a difference in the genetic background of the mice or the experimental conditions, including age, experimental protocols, time periods, and schedule.

Extinction and memory loss make it difficult for human patients to perform their daily activities (Bouwens et al., 2009). An important issue in clinical research is finding ways to overcome patient forgetfulness. 2-AG could be a drug target for treating dementias, such as Alzheimer’s disease—conditions in which forgetfulness, especially the loss of learned information, is one of the most common symptoms (Carlesimo and Oscar-Berman, 1992; Rogers and Friedman, 2008; Chen et al., 2012).

## Author Contributions

Y. Kishimoto, BC, MY, KS, MK designed research; Y. Kishimoto, BC, MY, JN performed research; Y. Kishimoto, BC, JN, Y. Kirino analyzed data; and Y. Kishimoto, BC, MK wrote the paper.

## Conflict of Interest Statement

The authors declare that the research was conducted in the absence of any commercial or financial relationships that could be construed as a potential conflict of interest.
